# Pharmacokinetics of Tamoxifen and Its Major Metabolites and the Effect of the African Ancestry Specific CYP2D6*17 Variant on the Formation of the Active Metabolite, Endoxifen

**DOI:** 10.3390/jpm13020272

**Published:** 2023-01-31

**Authors:** Comfort Ropafadzo Kanji, Georginah Nyabadza, Charles Nhachi, Collen Masimirembwa

**Affiliations:** 1 African Institute of Biomedical Science and Technology (AIBST), Harare P.O. Box 2294, Zimbabwe; 2 Department of Clinical Pharmacology, University of Zimbabwe College of Health Sciences, Harare P.O. Box 2294, Zimbabwe; 3 Sydney Brenner Institute for Molecular Bioscience (SBIMB), University of the Witwatersrand, Johannesburg 2000, South Africa

**Keywords:** tamoxifen, pharmacogenetics, CYP2D6*17, genetic polymorphism, pharmacokinetics, metabolism

## Abstract

Tamoxifen (TAM) is widely used in the treatment of hormone receptor-positive breast cancer. TAM is metabolized into the active secondary metabolite endoxifen (ENDO), primarily by CYP2D6. We aimed to investigate the effects of an African-specific *CYP2D6* variant allele, CYP2D6*17, on the pharmacokinetics (PK) of TAM and its active metabolites in 42 healthy black Zimbabweans. Subjects were grouped based on *CYP2D6* genotypes as CYP2D6*1/*1 or *1/*2 or *2/*2 (CYP2D6*1 or *2), CYP2D6*1/*17 or 2*/*17, and CYP2D6*17/*17. PK parameters for TAM and three metabolites were determined. The pharmacokinetics of ENDO showed statistically significant differences among the three groups. The mean ENDO AUC_0-∞_ in CYP2D6*17/*17 subjects was 452.01 (196.94) h·*ng/mL, and the AUC0-∞ in CYP2D6*1/*17 subjects was 889.74 h·ng/mL, which was 5-fold and 2.8-fold lower than in CYP2D6*1 or *2 subjects, respectively. Individuals who were heterozygous or homozygous for CYP2D6*17 alleles showed a 2- and 5-fold decrease in Cmax, respectively, compared to the CYP2D6*1 or *2 genotype. CYP2D6*17 gene carriers have significantly lower ENDO exposure levels than CYP2D6*1 or *2 gene carriers. Pharmacokinetic parameters of TAM and the two primary metabolites, N-desmethyl tamoxifen (NDT) and 4-hydroxy tamoxifen (4OHT), did not show any significant difference in the three genotype groups. The African-specific CYP2D6*17 variant had effects on ENDO exposure levels that could potentially have clinical implications for patients homozygous for this variant.

## 1. Introduction

Tamoxifen (TAM) has been the mainstay, adjuvant, and neoadjuvant treatment for estrogen receptor-positive (ER+) breast cancer for more than 40 years [1]. TAM is a selective oestrogen receptor modulator (SERM) that works by inhibiting estrogen binding at the receptor site, thereby inhibiting the hormone’s cell proliferation role. Five-year treatment with TAM in ER+ breast cancer patients has been observed to reduce the mortality rate by a third [2] and the recurrence rate by 30–50% [3]. However, about 30–50% of women on TAM therapy experience disease recurrence [4,5]. Determinants for TAM treatment efficacy includes genetics [6], drug-drug interactions [7], and poor treatment compliance [8].

TAM is a prodrug that requires extensive metabolism via the cytochrome P450 (CYP) enzymes to its metabolites N-desmethyl tamoxifen (NDT), 4-hydroxy tamoxifen (4OHT) and endoxifen (ENDO) to elicit anti-tumor activity [9]. The metabolites 4OHT and ENDO have 100-fold more affinity for the target oestrogen receptor and 30-to-100-fold more potency than TAM the parent drug. The secondary metabolite of TAM, ENDO, which is responsible for most of TAM’s anti-tumour effects, is mainly produced by the action of CYP2D6 on *NDT* [10,11]. Genetic variants in the gene coding for the CYP2D6 enzyme may lead to reduced enzyme activity (12). CYP2D6 activity determines ENDO levels, and genetic variants or drug interactions that affect CYP2D6 activity affect the ENDO exposure levels in vivo (13). Several studies have observed impaired formation of ENDO in carriers of CYP2D6 null or reduced function enzyme variants [12,13,14].

Drug-gene or drug-drug interactions may influence TAM treatment outcomes [15]. The role of TAM pharmacogenetics in breast cancer treatment outcomes has been extensively studied in Caucasian populations [16] The majority of the studies found that patients with the poor CYP2D6 metabolizer status (PM) responded poorly to TAM. The observed poor response was due to reduced capacity to produce the active metabolite ENDO [14,17,18]. The CYP2D6*4 variant is associated with majority of PM enzyme status in Caucasians, with a frequency of 20% [19]). This variant is less prevalent in Asian and African populations, where its frequency distribution is less than 2% [20]. However, Asian populations have a high prevalence of the low activity variant CYP2D6*10, which confers reduced metabolic activity. The frequency of CYP2D6*10 in the Asian population is 41.17% [21]. 

Seven [5,22,23,24,25] retrospective studies of patients of Asian ethnicity, reported the association of the *CYP2D6* genotype with clinical outcomes in Asian populations. CYP2D6*10 is the major variant in the Asian population. Four of the seven studies reported a significant reduction in progression-free survival, disease-free survival or time to disease progression odds ratio in patients homozygous for CYP2D6*10 compared with wild-type homozygotes. Recent studies in Japanese patients have demonstrated the potential utility of this knowledge to be applied in dose adjustment, where the dose of TAM was increased from 20 mg/day to 30 and 40 mg/day with improved efficacy and no apparent increase in adverse drug effects. The higher dose led to the intermediate metabolizer’s (IM) patients producing the active metabolite, ENDO, comparable to that produced by extensive metabolizers when given the standard dose of 20 mg/day. 

CYP2D6*4 and CYP2D6*10 variants have been observed at very low to intermediate frequencies in African populations, with reported allele frequencies ranging from 0–12% for CYP2D6*4 and 0–19% for CYP2D6*10 [26,27,28,29]. However, African populations have a high frequency of a *CYP2D6* variant unique to people of African origin, CYP2D6*17, that exists at a frequency of more than 34% in these populations [30]. As there are limited human in vivo single-dose studies assessing the effect of CYP2D6*17 carrier status on the metabolism of TAM, we conducted a single-dose pharmacokinetic study to determine the effect of the CYP2D6*17 variant on the pharmacokinetics of TAM and its metabolites. 

## 2. Materials and Methods

### 2.1. Trial Design

**Figure 1:**
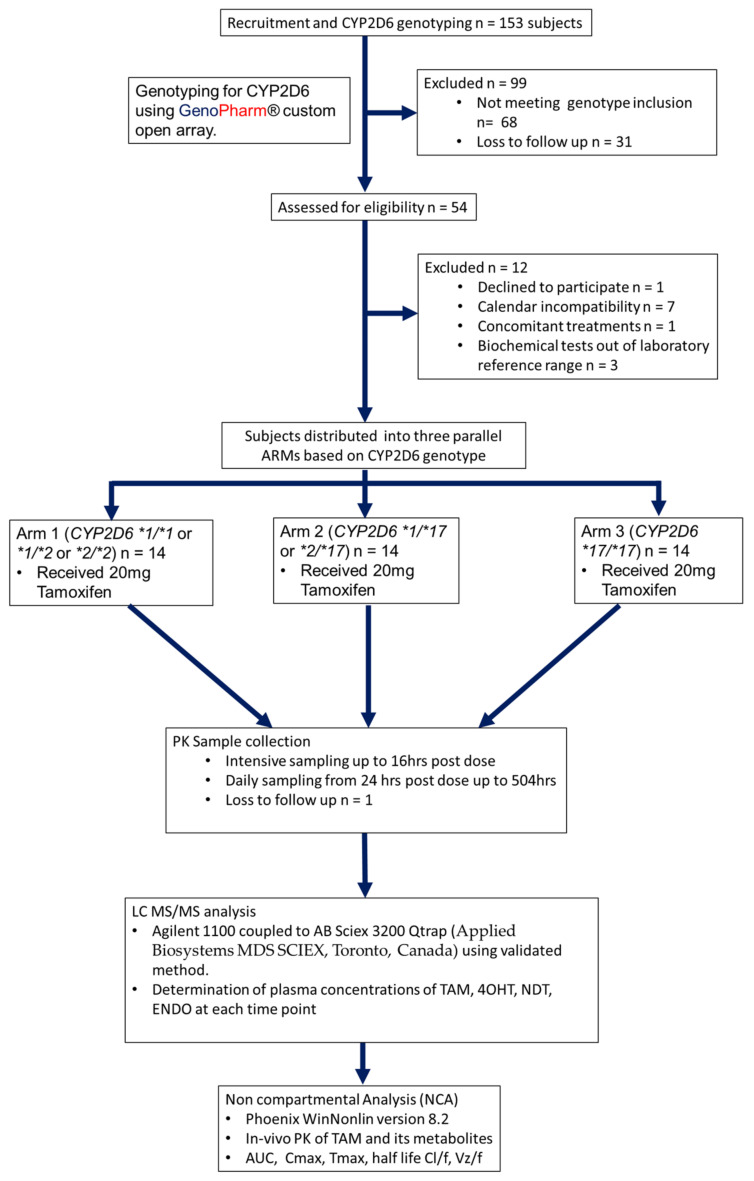
Study design and sequence flow chart for the pharmacokinetic study to determine the effect of CYP2D6*17 on the metabolism of TAM to ENDO.

We conducted an open label, three parallel arms, single treatment, single oral dose clinical study in healthy subjects at the African Institute of Biomedical Science and Technology, Harare, Zimbabwe. The study design and workflow are shown in Figure 1. Forty-two eligible, healthy males and females were enrolled sequentially between April 2019 and August 2021. Participant demographics are presented in Table 1. Each individual signed an informed consent form. The study was approved by the Medical Research Council of Zimbabwe (MRCZ) and the Medicines Control Authority of Zimbabwe (MCAZ). Two weeks prior dose administration until study completion, subjects were not allowed to smoke, take alcohol, take prescribed or over the counter medication, or recreational drugs. During the screening process, blood samples were obtained to determine the *CYP2D6* genotype. Subjects eligible for inclusion in the study were carriers of the following *CYP2D6* genotypes: CYP2D6*1/*1 or *1/*2 or *2/*2 (CYP2D6*1 or *2), CYP2D6*1/*17 or *2/*17 and CYP2D6*17/*17. Subjects were grouped equally into three arms of 14 subjects based on the *CYP2D6* genotypes: Arm1 *1/*1 or *1/*2 or *2/*2, Arm 2 *1/*17 or *2/*17 and Arm 3 *17/*17. Each participant received a single oral dose of 20 mg TAM. Blood samples were collected prior to the dose, followed by extensive sampling in the first 24 h, during which subjects were housed at the clinical trial unit for 44 h. The subjects were housed at the clinical trial unit from 18 h before dose administration to 26 h after administration. Daily sampling at 24-h intervals until 504 h post-dose administration was conducted were subjects visited the clinical trial unit daily. This resulted in a total of 34 samples per participant. Blood samples were collected to measure the plasma concentrations of TAM and its metabolites.

### 2.2. CYP2D6 Genotype

A 4-mL blood sample was collected in an EDTA tube (standard clinical purple-top tube) and kept on ice. Within 1 h of collection, the samples were processed and aliquots stored at −20 °C until analysis. The *CYP2D6* genotype for the *1 (wild-type) or *2(rs16947, normal activity) and *17 (rs28371706, reduced activity) was determined using TaqMan chemistry on the GenoPharm^®^ custom open array. Supplementary Table S1 contains the variants and SNP ids that were investigated. In brief, the CYP2D6 enzyme was tested for genetic variation. As per manufacturers protocol DNA was extracted from 200 µL of peripheral whole blood using the MagMAX™ DNA Multi-Sample Ultra 2.0 Kit on the Thermofisher KingFisher™ Flex Purification System with the MagMAX Ultra 2.0–200 µL script for KingFisher Flex. Extracted DNA was quantified with the Qubit 4 fluorometer using the Qubit dsDNA BR Assay Kit and stored at −20 °C short term before analysis. Genotyping for *CYP2D6* was performed on the GenoPharm^®^ custom open array panel as per the manufacturer’s protocol. In brief, a reaction mixture of 5 µL genomic DNA and 5 µL of TaqMan™ Genotyping master mix (Cat. No. 4462164) was prepared per sample. The PCR mix was transferred to the GenoPharm^®^ custom open array panel using the automated Applied Biosystems™ QuantStudio™ 12K Flex OpenArray™ AccuFill™ System according to the manufacturer’s instructions. A no template control (reaction mixture with all reagents but no template DNA) was included in each run. The 33 nl reaction mix was run per data point on the Applied Biosystems™ QuantStudio™ 12K Flex Real-Time PCR System (Thermo Fisher Scientific, Marsiling Industrial Estaste, Singapore). Genotypes for the samples were determined by the TaqMan™ Genotyper Software as per the manufacturer’s instructions. Genotype calls were generated with TaqMan^®^ Genotyper Software. The *CYP2D6* copy number was determined using the Applied Biosystems TaqMan copy number assays for exon 9, the primary copy number assay (Assay ID: Hs00010001_cn) to quantify *CYP2D6* duplications or identify *CYP2D6* gene deletions (CYP2D6*5) in the samples. AlleleTyper™ software was used to convert sample genotype information for the CYP genes interrogated to the star (*) allele nomenclature using a predefined allele translation table that maps a specified allele pattern to the star allele call. 

### 2.3. Measurement of Plasma Tamoxifen and Metabolite Concentrations

At each sampling time point, a 4-mL blood sample was collected in an EDTA tube (standard clinical purple-top tube) and stored on ice. The following time points were used in this study, 0, 0.5, 1, 1.5, 2, 2.5, 3, 4, 5, 6, 8, 12, 16, 24, 48, 72, 96, 120, 144, 168, 192, 216, 240, 264, 288, 312, 336, 360, 384, 408, 432, 456, 480 and 504 h. Within 1 h of collection, the blood was centrifuged (3000 rpm for 10 min at 4 °C), and the plasma was isolated and stored at −80 °C. 

### 2.4. Bioanalysis of Tamoxifen and Metabolites

Plasma samples were extracted using protein precipitation with ice cold acetonitrile as the extraction solvent. Briefly, 200 µL of plasma was spiked with 10 µL of 2 µg/mL propranolol (internal standard) followed by addition of 590 µL of ice-cold acetonitrile. The mixture was vortexed for 30 s, sonicated for 2 min before centrifugation at 16,000× *g* for 10 min. A volume of 700 µL of supernatant was collected and was evaporated to dryness under a gentle stream of nitrogen. The dried residue was reconstituted in 50 µL of mobile phase and 10 μL was injected into the LC/MS-MS for analysis.

### 2.5. LC-MS/MS Conditions

A 3200 Q TRAP Series triple quadrupole (Applied Biosystems MDS SCIEX, Toronto, Canada) liquid chromatography- mass spectrometry (MS/MS) system coupled to an Agilent 1100 series HPLC system (Agilent Technologies, Waldbronn, Germany) was used to carry out all the analysis operated using Analyst software version 1.6 (AB SCIEX, Toronto, Canada). The compounds of interest were separated using Zorbax C18 2.1 X100 mm, 3.5 µm column (Zorbax Agilent, Santa Clara, CA, USA). The mobile phase consisted of 0.1% formic acid in 10mM ammonium formate solution as mobile phase A and 0.1% formic acid in acetonitrile as mobile phase B delivered using a gradient elution: 0–9 min, B 30%, 9.01–9.5 min, B 52%- and 9.5–13-min B 30%. The column was maintained at a temperature of 40 °C. 

Analytes were followed using multiple reaction monitoring (m/z 372.5→ 72.2, 374.4→ 58.1, 358.4→ 58.0, 388.4→ 72.2 and 260.3→ 183.3 for TAM, ENDO, NDT, 4OHT and propranolol as internal standard (IS) respectively). MS/MS analyses were performed in positive ionization mode, ion source temperature 500 °C, curtain gas 25, ion spray voltage 5500, GS1 and GS2 gas 50 and 30 respectively. The lower limit of quantification was 0.05 ng/mL for 4OHT and 0.1 ng/mL for TAM, ENDO and NDT with the standard curve linear in the range between 0.01–1000 ng/mL.

### 2.6. Pharmacokinetic Analysis

Pharmacokinetic parameters were estimated from plasma concentrations using Non compartmental analysis (NCA) in WinNonlin software version 8.2 (Certara). The area under the curve (AUC) from time of dosing to the last quantifiable concentration (AUC_last_) and infinity (AUC_0-∞_) was estimated using the linear and logarithmic trapezoidal rule. The linear up and log down method was used. The elimination rate constant (*K*el) was determined by the program using nonlinear regression of the natural logarithm of concentration values in the elimination phase. The terminal half-life (T_half_) was calculated using the equation T_half_ = ln2/λ. The apparent clearance, (CL/F) was determined from the equation CL/F = Dose/AUCinf. PK profiles were plotted as graphs of TAM and metabolites concentrations vs. time.

TAM dose increase estimation to predict the ENDO steady-state plasma concentrations (C_ss_) and achieve therapeutic levels of ENDO in patients carrying the CYP2D6*17/*17 was done in Phoenix WinNonlin using the non-parametric superposition tool. As per Phoenix WinNonlin users guide, the non-parametric superposition object in Phoenix is based on non-compartmental results representing single-dose data in order to forecast drug concentrations after numerous doses at steady state. The predictions are based on an accumulation ratio estimated from the terminal slope, which can be utilized for simple (constant dose) or elaborate dosing plans (based on the Phoenix WinNonlin User’s Guide). The simulated TAM dose was increased from 20 mg/day to 30 mg/day to finally 40 mg/mL.

### 2.7. CYP2D6*17 Activity Score and Calibration Curve 

Metabolic ratio (MR) for NDT to ENDO were determined as concentration of ENDO divided by concentration of NDT. Standard curve for the activity as (MR) of ENDO/NDT vs. predicted activity score based on the consensus activity score was performed using external calibration as reported by L. Thorén and colleagues [12]. Linearity was assessed by linear regression of the calibration curve. Metabolic ratios from this study were used to interpolate the predicted activity score.

## 3. Statistical Analysis

To detect a 25% difference in the Cmax AUC_0-∞_ of ENDO between CYP2D6 normal metabolizer (NM) phenotype and CYP2D6 IM phenotype, with a two-sided 5% significance level, power of 80%, and allowing 20% subject dropout. The study required 14 subjects per group. This was based on a within-patient variation of 35% in the pharmacokinetics of TAM and ENDO. The Schumann’s two, one-sided *t* test was used for sample size determination.

Statistical analysis was performed using the SPSS^®^ software package, version 22.0 (IBM, North Castle, NY, USA) and visualization in GraphPad prism version 8.4.3. Analyses included descriptive statistics, paired *t* tests. Analysis of variance (ANOVA) was performed on the AUC and C_max_ after transformation of the data to their natural logarithmic (ln) values.

## 4. Results

### 4.1. Baseline Characteristics

A total of 42 subjects were enrolled in the study, 3 females and 39 males as shown in Table 1. Out of the initial 153 subjects screened for *CYP2D6* genotype 85 subjects were eligible. From the 85 subjects, 31 were lost to follow up, 54 were screened for study eligibility and 42 were dosed with single oral dose of 20 mg TAM. No concomitant medicines were taken by the subjects. Amongst the three *CYP2D6* genotype groups, there were no significant differences in participant demographic characteristics (data not shown). TAM was generally well tolerated by the subjects across all groups and there were no significant differences in physical evaluation, vital signs or lab tests observed.

### 4.2. Pharmacokinetic Analysis

**Figure 2:**
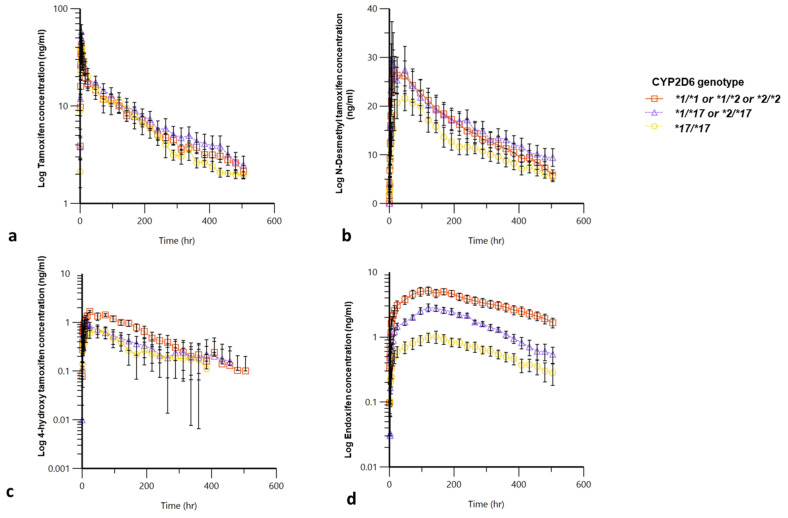
The PK profiles are for the three genotype groups (CYP2D6*1 or *2, CYP2D6*1/*17 or *2/*17, and CYP2D6*17/*17) showing log transformed plasma concentration versus time for (**a**) TAM, (**b**) NDT, (**c**) 4OHT and (**d**) ENDO after single oral 20 mg dose of TAM.

Mean plasma concentration time profiles following oral administration of 20 mg TAM are shown in Figure 2. TAM was rapidly absorbed and was quantifiable at 0.5 h post dose administration with mean T_max_ at 4.25 h. PK parameters of TAM and metabolites are summarized in Table 2. TAM shows inter individual variation in the three groups however there was no significant difference in the mean PK parameters of TAM among the three studied CYP2D6 groups all (*p* > 0.05). The mean ENDO AUC_0-∞_ of 452.01 (196.94) hr·ng/mL, in CYP2D6*17/*17 subjects and AUC_0-∞_ 930.69 (212.41) h·ng/mL in CYP2D6*1/*17 or *2/*17, subjects were 5.8-fold and 2.8-fold lower than in CYP2D6*1 or *2 subjects with AUC_0-∞_ of 2625.5(1167.68) h·ng/mL. This observed difference in AUC was statistically significant (*p* < 0.001). A similar trend was observed for mean C_max_, where we observed a 5-fold and 2-fold statistically significant difference for C_max_ in CYP2D6*1 or *2 subjects compared to CYP2D6*17/*17 and CYP2D6*1/*17 (*p* < 0.001) respectively. Analysis by *CYP2D6* genotype, for the primary metabolites NDT showed no difference between the different groups. Table 2 shows that the presence of CYP2D*17 resulted in a statistically significant difference in Cmax and T_max_ when compared to CY2D6*1 or *2 (*p* < 0.05). However, there was no statistically significant difference for 4OHT AUC within the three study arms.

### 4.3. Activity Score for CYP2D6*17

**Figure 3:**
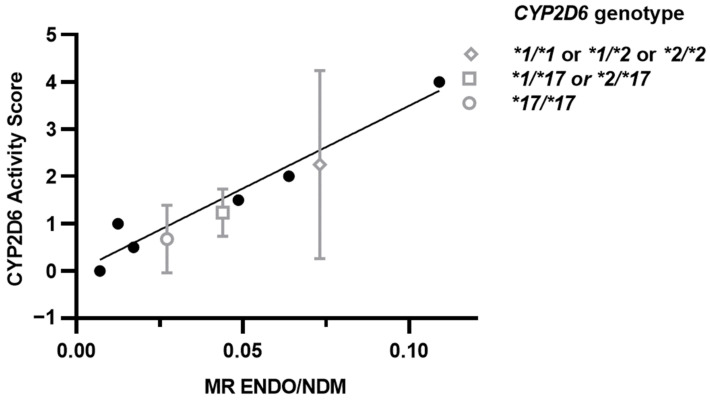
CYP2D6 activity calibration curve plotted as observed MR [12] vs. activity score obtained from the CPIC guidelines [32]. The interpolated predicted activity scores for the three arms are presented on the graph as the median and interquartile range (IQR). The predicted activity score for our study is shown in grey for CYP2D6*1 or *2, CYP2D6*1/*17 or *2/*17, and CYP2D6*17/*17. Predicted activity scores on the y axis were based on the current CPIC guidelines.

CYP2D6 mediates the major steps in the formation of ENDO from NDT. We therefore investigated the relationship between *CYP2D6* genotypes and the MR of ENDO/NDT. The mean concentrations were significantly different between genotype groups (*p* < 0.05) showing increased MR with CYP2D6 activity despite considerable interindividual variability between the different arms (Figure 3). Using the ENDO/NDT MR to capture the role of CYP2D6 for allele variants *17/*17 and *1/*17 and *2/*17 on the calibration curve we interpolated the predicted activity score. The median interpolated predicted activity score for homozygous CYP2D6*17 was 0.667 vs. the activity score of 1 in the current CPIC current guidelines as shown in Figure 3. Therefore, the activity score of CYP2D6*17 was estimated at 0.334. This is lower than the activity score of 0.5 in the current guidelines [31,32].

### 4.4. Dose Escalation and Predicted Endoxifen C_ss_

**Figure 4:**
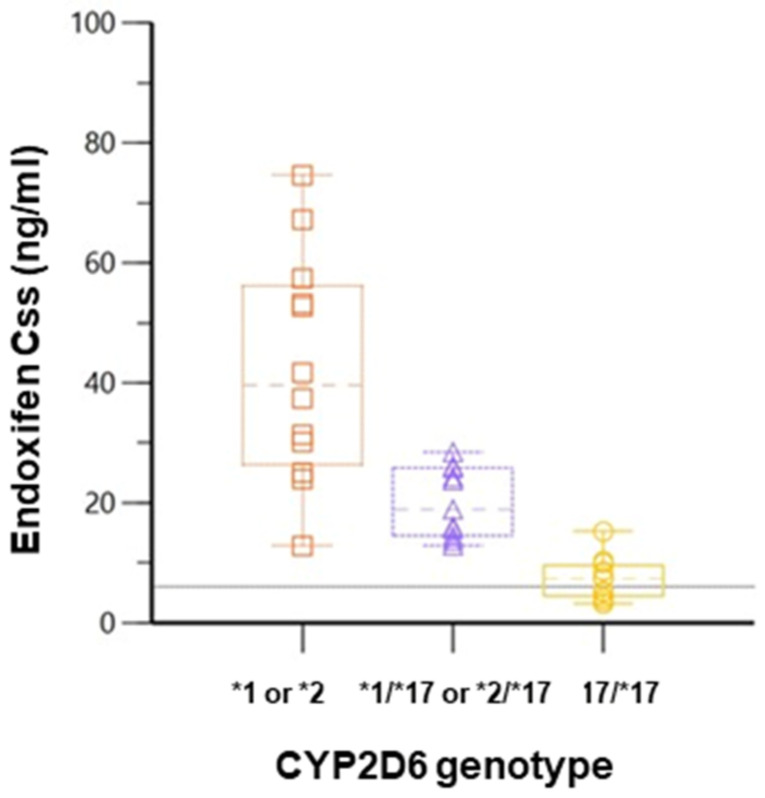
Simulated steady state concentrations of ENDO in *CYP2D6* genotype arms, with horizontal line highlighting ENDO therapeutic threshold as reported by Madlensky [14]. Boxes interquartile range (IQR) including median.

As shown in Figure 4, the simulated median ENDO C_ss_ in arms 1 and 2 was higher than the reported ENDO therapeutic threshold of 5.9 ng/ml [14]. However, 42% of subjects in the homozygous CYP2D6*17 arm were below the threshold, with a group median of 7 ng/mL.

**Figure 5:**
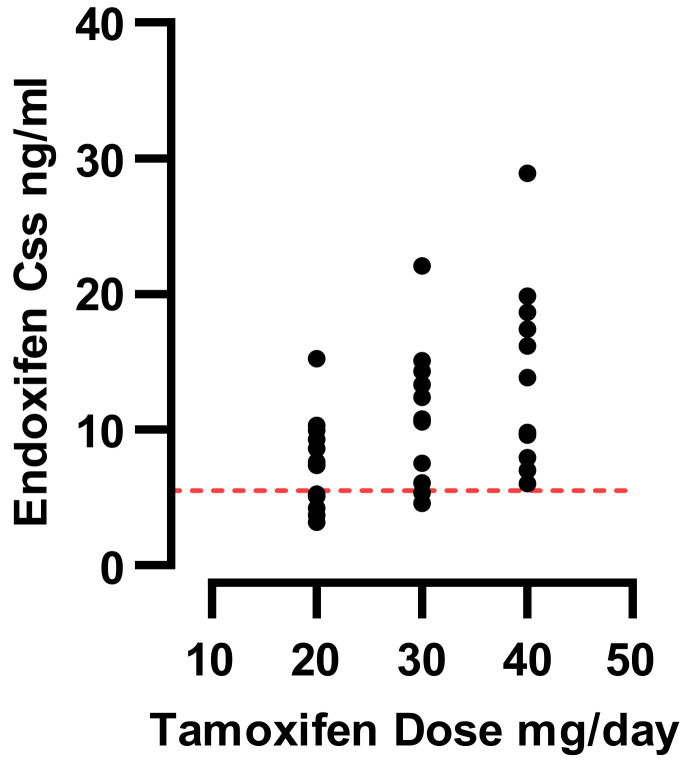
Dose escalation simulation plot of TAM from 20 to 40 mg/day with predicted change of mean steady state ENDO concentration in the CYP2D6*17/*17 arm. The dotted red line shows putative reported ENDO therapeutic threshold at 5.97 ng/mL.

Simulating TAM dose escalation in the CYP2D6 *17/*17 arm from 20 to 30 and 40 mg/day resulted in a significant increase in the median plasma ENDO concentration. At a dose of 30 mg/day, n = 12 subjects were above the putative therapeutic ENDO threshold of 5.97 ng/mL, and a further increase in TAM dose to 40 mg/day resulted in all 14 subjects in the homozygous CYP2D6*17 arm having ENDO concentrations above the putative ENDO threshold of 5.97 ng/mL. The mean ENDO concentrations in the simulated dose escalation are shown in Figure 5.

## 5. Discussion

TAM is important as an adjuvant or neoadjuvant in the management of oestrogen receptor positive breast cancer [33]. The role of pharmacogenetics on the pharmacokinetics and efficacy of TAM have been subjects to extensive research efforts [34,35]. Multiple enzymes are involved in TAM biotransformation, suggesting that there could be more than one enzyme polymorphism that can influence the pharmacokinetics of TAM [36]. Several TAM pharmacogenetic studies have been published in breast cancer patient cohorts [37] but to our knowledge, this is the first pharmacogenetic study evaluating the effect of CYP2D6*17 on the pharmacokinetics and metabolism of TAM and its metabolites in a controlled black healthy subjects’ population. 

TAM metabolism involves several CYP enzymes that include but not limited to CYP2B6, CYP2C19, CYP3A4, CYP3A5, and CYP2D6 [36] and CYP mediated metabolism pathways are subject to genetic variability that can affect exposure levels of TAM and metabolites. Genetic variation in CYP3A4, CYP3A5, and CYP2C19 may also affect TAM metabolism. However, it is less clear how these enzymes influence ENDO plasma levels [38,39,40,41,42]. Studies have shown that variability in *CYP2D6* results in different exposure levels of ENDO between wild type and variant alleles, Studies have reported a gene dose effect for *CYP2D6* and ENDO formation for patients on TAM therapy [43]. 

In this study, TAM pharmacokinetics showed high interindividual variability and did not show any statistically significant differences in TAM PK parameters across the 3 different *CYP2D6* genotype arms. TAM was rapidly absorbed, with a mean T_max_ of 4.25 h. A high overall mean C_max_ of 55 ng/mL was observed, which is comparable to what has been reported by Adam et al. [44] of 42 ng/mL, and earlier studies with radio labelled TAM showed peak TAM concentrations ranging from 60–100 ng/mL [45]. The observed long terminal half-life of 6.6 days was within the reported TAM half-life range of 5–9 days [44,45,46]. As reported by Etienne [47] all the three studied metabolites were detectable from a single 20 mg dose of TAM. NDT was quantitatively the predominant metabolite and 4OHT was quantitatively the minor metabolite. This agrees with what has already been published, where NDT is a product in the major metabolic pathway of TAM metabolism [47]. NDT had a higher AUC than the parent drug as has been observed before in a bioequivalence study in female volunteers [48]. In general, the major metabolites NDT and ENDO had longer half-lives than TAM, indicating increased exposure and circulation duration of the metabolites.

Previous studies have shown that CYP2D6 activity accounts for 39–58% of ENDO interindividual variability [49,50,51]. In this study, we have described an association between expression of CYP2D6*17, a reduced activity enzyme variant, and pharmacokinetic parameters of TAM and its major active metabolite, ENDO. The CYP2D6*17 allele has a clear impact on ENDO pharmacokinetics in homozygous carriers, resulting in a 5.8-fold reduction in AUC_0-∞_ and 5-fold reduction in C_max_ in homozygous individuals compared to the wild type. In a study of TAM-treated Algerian breast cancer patients, including those genotyped as CYP2D6*17/*17. The median ENDO concentration for IMs was 2.4 times lower than that of CYP2D6*1 carriers, clearly showing that the reduced activity variant in the homozygous state results in reduced levels of ENDO compared to the NM and ultra-rapid metabolizer phenotypes [52]. 

The debate on the clinical relevance of the TAM-*CYP2D6* drug-gene interaction has not yet been resolved as there are conflicting results from different studies on the role of the *CYP2D6* genotype on the clinical outcomes of breast cancer treatment. Recent studies [14,18] have suggested a plasma ENDO therapeutic threshold of 5.9 ng/mL, which results in improved treatment outcomes by reducing the risk of breast cancer recurrence by 30% [14]. About 40% of CYP2D6*17 homozygous subjects in our study were below the reported putative therapeutic threshold for ENDO. Demonstrating that some patients homozygous for CYP2D6*17 will still achieve therapeutic levels of ENDO whilst others would be below the therapeutic range. Studies have shown that patients below this threshold could be at a higher risk of disease relapse or death. Thus, in clinical practice, clinicians should consider a dose increase in patients carrying the CYP2D6*17 variant who do not respond to standard dosing of 20 mg daily of TAM. A dose increase would reduce the potential risk of therapeutic failure, as what has been observed in the clinical setup, where PM patients with low ENDO levels had higher risk of disease recurrence [5,40,53,54,55].

Several dose escalation studies with individuals with reduced CYP2D6 metabolic activity, such as the CYP2D6*10, have shown that individuals with sub-therapeutic plasma ENDO levels can achieve therapeutic effective ENDO concentrations by increasing TAM doses from 20 to 40 or 60 mg [56,57,58,59]. With dose escalation, a linear increase in ENDO plasma concentrations has been observed in some studies within the 20 to 60 mg/day TAM dose range [56,57,60]. We simulated dose increases in IM subjects homozygous for CYP2D6*17 based on the assumption of linear PK. The simulation indicated that doses of 40 mg/day would ensure that all subjects homozygous for CYP2D6*17 would reach the proposed putative threshold of 5.97 ng/mL. Our results align with what has been reported by Puszkiel and colleagues, who, using a population pharmacokinetic (PopPK) model, showed that *CYP2D6* IMs and PMS would require a dose increase of 40 and 80 mg per day, respectively [61]. Genotyping of clinically significant *CYP2D6* alleles and subsequent dose adjustment in IM and PM patients has been observed to result in increased ENDO plasma levels [59]. However, some have proposed the use of therapeutic drug monitoring for ENDO to determine the dose adjustment [62,63]. Some studies have used sub-therapeutic baseline ENDO concentrations to determine the dose of TAM. The dose adjustments resulted in increased levels of END and improved treatment outcomes [51,60]. However, despite several dose-escalation attempts, it is important to note that increasing TAM dose has been shown to benefit IM patients more than PM phenotype patients in terms of increasing ENDO plasma levels [51,58,60]. Dezentjé and colleagues used both ENDO concentration and *CYP2D6* genotype in a dose escalation study that resulted in all patients, including PMs, having a concentration above the threshold of 5.97 ng/mL [64]. More studies need to be done to reach a consensus on how best to use *CYP2D6* genotypes as predictors for patients who can benefit from dose adjustment.

TAM metabolism is complex, involving several enzymes that can be induced or inhibited. This can result in CYP-mediated drug-drug interactions that can influence the levels of TAM and its metabolite. Current CPIC guidelines for TAM therapy highlight the need to avoid moderate and strong CYP2D6 inhibitors [32]. Binkhorst and colleagues demonstrated that strong CYP2D6 inhibitors such as paroxetine and fluoxetine reduced ENDO to sub-therapeutic levels, posing a risk for poor treatment outcomes in patients, including those who are CYP2D6 NMs [65]. Rifampicin, a very strong CYP450 enzyme inducer was shown to result in reduced plasma levels of TAM and its metabolites, including ENDO. This phenomenon was explained by the ability of rifampicin to induce CYP3A4 and potentially UDP-glucuronosyltransferase (UGT) [66], resulting in increased clearance of ENDO, as UGTs play a pivotal role of converting ENDO to an inactive glucuronide metabolite. Hence, due to the drug interactions with TAM, there is a risk of enzyme phenocopying, as has been observed where NM phenotypes after taking CYP2D6 inhibitors had low ENDO exposure levels that were comparable to PM and IM phenotypes. However, a recent study found that taking probenecid with TAM resulted in an increase in ENDO exposure levels, even in PM patients with limited or no side effects. This reported drug-drug interaction could be important in increasing the exposure levels of ENDO in IM and PM patients who have been observed to have sub-therapeutic ENDO levels. The observed TAM-probenecid drug interaction was proposed to be through CYP induction and inhibition of the glucuronidation pathway [67] Such interactions if proved to have no or low adverse drug effects with long-term repeated use, could be useful as boosters, as the case with ritonavir in Antiretroviral therapy for HIV patients [68].

When the activities of CYP2D6 variants were compared in vitro, CYP2D6*17 activity was less than 15% that of CYP2D6*1 [69]. Bodies such as the Clinical Pharmacogenetics Implementation Consortium (CPIC), the Dutch Pharmacogenetics Working Group (DPWG), and the Canadian Pharmacogenomics Network for Drug Safety (CPNDS) have come up with pharmacogenetic guidelines to assist with dosing for patients on TAM therapy [32,70]. CPIC and DPWG guidelines have assigned an activity score of 0.5 to the CYP2D6*17 variant [71]. In this study, however, we predicted a lower activity score value of 0.34 with respect to the CYP2D6*17 allele and TAM metabolism. Efforts to understand the functional status of CYP2D6*17 have demonstrated that CYP2D6*17 activity is substrate-specific, and the calculated activity score values ranged from 0.09 to 0.54 with different CYP2D6 substrates [32,72,73]. A recent study [74] reported comparable in vitro activity between CYP2D6*17 and CYP2D6*10. This review article highlighted that the CYP2D6 activity was below the assigned activity score value of 0.5 [74]. The lower value we predicted underscores the potential need to re-evaluate the activity scoring of CYP2D6*17 with TAM to accurately capture the phenotype group, as there is a risk of underestimating the necessity for dose adjustment and reducing the predictive performance of *CYP2D6*. Re-evaluation of the CYP2D6*10 variant from the previous activity score value of 0.5 to 0.25 resulted in improved prediction of ENDO by *CYP2D6* in Asian populations [50].

### Limitations

Limitations of our study include having more male subjects (93%) compared to females, yet >98% of breast cancer is female breast cancer; hence, most patients who are prescribed TAM are female breast cancer patients. TAM is still an important drug for hormonal therapy in the treatment of ER+ male breast cancer [75]. Pharmacokinetics has been shown to differ between male and female patients due to different physiological variables. Gender has been associated with differences in steady state endoxifen concentrations [76], but the clinical significance of gender has not been fully established [35]. The second limitation is that our evaluation on the effect of *CYP2D6* variants was on a single dose administration on a drug that is given on a daily basis for up to 5-years. However, we performed dose simulations to predict steady-state plasma levels of ENDO. The study controlled for the demographic parameters, resulting in demographic homogeneity for age, weight, and BMI, which may partially explain the lack of associations between plasma levels and the demographic parameters of age, weight, and BMI. Thirdly the study had a small number of subjects. These findings should therefore be confirmed in different cohorts with a larger number of subjects. 

## 6. Conclusions

The presence of the CYP2D6*17 allele in a homozygous state can significantly influence the generation of ENDO from TAM. Our study shows statistically significant findings that the reduced activity variant CYP2D*17 is associated with low levels of the active metabolite ENDO. Through simulated dose escalations, it further shows that increasing the dose of TAM to 40 mg/day in subjects homozygous for CYP2D6*17 would result in all the subjects attaining therapeutic levels of ENDO that are above the putative 5.97 ng/mL threshold. Our study proposes a revised activity score demonstrating a lower capacity to generate ENDO. Owing to the high prevalence of the CYP2D6*17 variant among the black Zimbabwean population, the results of this study might provide benefit for breast cancer patients upon the introduction of personalised TAM therapy in Zimbabwe. Further studies are warranted to confirm our findings. 

## Figures and Tables

**Table 1:** Baseline characteristics of subjects enrolled in the study.

Participant Baseline Characteristics		
	N ^1^	42
Sex	Female (N, [%])	3 [7]
	Male (N, [%])	39 [93]
	Race	Black
	Age: years (median, [range])	23.4 [19.1–30.2]
	Weight: kg (median, [range])	65.0 [53–89]
	Height: cm (median, [range])	173.0 [155–192]
	BMI: kg/m^2^ (median,] [range])	22.5 [19.2−26.7]

^1^ N is the number of subjects.

**Table 2:** Effect of *CYP2D6* genotypes on the Pharmacokinetics of TAM and its main metabolites following a single oral dose administration of 20 mg TAM.

	CYP2D6*1/*1, *1/*2 or *2/*2	CYP2D6*1/*17 or *2/*17	CYP2D6*17/*17	*p Value*
*Tamoxifen*				
*C_max_ (ng/mL)*	51.88 (18.25)	62.96 (25.68)	54.23 (14.13)	0.3803
*T_max_ (h)*	4.154 (1.97)	3.94 (1.27)	4.67 (2.22)	0.0503
*AUC_0-last_ (h·ng/mL)*	3650.75 (1203.75)	4246.94 (2068.27)	3520.99 (1874.01)	0.5116
*AUC_0-∞_ (h·ng/mL)*	4560.56 (2030.04)	4853.29 (2285.58)	4673.49 (2036.21)	0.9345
*T_half_ (h)*	152.81 (44.15)	160.90 (34.58)	165.27 (60.73)	0.7834
*V/f (L)*	1530.42 (833.69)	1166.25 (585.18)	1344.75 (790.65)	0.4404
*CL (L/h)*	4.88 (1.38)	4.84 (1.90)	4.97 (1.84)	0.9793
*N-desmethyl tamoxifen*				
*C_max_ (ng/mL)*	33.98 (16.01)	39.66 (20.33)	31.37 (9.447)	0.3791
*T_max_ (h)*	39 (27.09)	25.25 (19.15)	49.78 (31.44)	0.0593
*AUC_0-last_ (h·ng/mL)*	8542.90 (2863.16)	7742.71 (2072.57)	7232.27 (1387.74)	0.2918
*AUC_0-∞_ (h·ng/mL)*	10,680.76 (4546.64)	9944.45 (2567.0)	9372.26 (2088.38)	0.5693
*T_half_ (h)*	262.00 (114.97)	297.06 (179.08	256.24 (125.26)	0.7165
*V/f (L)*	666.231 (299.65)	578.21 (353.64)	603.4 (240.53)	0.7306
*CL (L/h)*	2.30 (1.04)	1.79 (0.4)	2.39 (0.55)	0.0701
*4* *-* *Hydroxy-* *tamoxifen*				
*C_max_ (ng/mL)*	1.518 (0.57)	1.06 (0.41)	0.724 (0.360	0.0002 *
*T_max_ (h)*	29.33 (14.89)	24 (16.49)	44.8 (28.06)	0.0340 *
*AUC_0-last_ (h·ng/mL)*	245.33 (142)	218.72 (153.63)	132.318 (121.7)	0.0950
*AUC_0-∞_ (h·ng/mL)*	321.067 (129.19)	299.35 (168.81)	245.27 (170.76)	0.4305
*T_half_ (h)*	98.57 (79.17)	122.99 (50.12)	103.14 (65.24)	0.6036
*V/f (L)*	4229.37 (2072.66)	4629.26 (1752.04)	30,269.62 (7873.91)	<0.001 *
*CL (L/h)*	103.64 (32.79)	128.48 (69.80)	288.16 (257.62)	0.070
*Endoxifen*				
*C_max_ (ng/mL)*	5.26 (2.14)	2.95 (1.0)	1.034 (0.50)	<0.001 *
*T_max_ (h)*	124.8 (40.8)	132 (18.14)	144 (26.83)	0.2517
*AUC_0-last_ (h·ng/mL)*	1857.62 (854.39)	808.42 (180.82)	355.51 (132.36)	<0.001 *
*AUC_0-∞_ (h·ng/mL)*	2625.5 (1167.68)	930.69 (212.41)	452.01 (196.94)	<0.001 *
*T_half_ (h)*	246.05 (102.01)	171.87 (35.49)	243.71 (99.46)	0.0508
*V/f (L)*	3324.79 (1825)	3940.09 (2330.34)	11,931.32 (4261.43)	<0.001 *
*CL (L/h)*	12.57 (9.99)	20.35 (6.2)	38.6 (17.63)	<0.001 *

Data represented as mean (standard deviation). Analysis of variance (ANOVA) was used to test for the differences between the means of the key pharmacokinetic (PK) parameters: area under the plasma concentration–time curve (AUC), peak plasma concentration of the compound (Cmax), time needed to achieve Cmax (T_max_), T_half_, elimination half-life, hours (hr), clearance (CL), the apparent volume of distribution (V/f), AUC0-last, area under the plasma concentration–time curve from time zero to the last sampled time point; AUC0-∞, AUC from time zero to infinity. * Statistically significant difference is defined as *p* < 0.05.

## Data Availability

The data that support the findings of this study are available upon reasonable request.

## Supplementary Materials

The following supporting information can be downloaded at https://www.mdpi.com/article/10.3390/jpm13020272/s1, Table S1: *CYP2D6* SNP variants that were tested to determine the subjects *CYP2D6* genotypes prior to dosing.
