# Supplementary material for: Pharmacokinetics of Tamoxifen and Its Major Metabolites and the Effect of the African Ancestry Specific CYP2D6*17 Variant on the Formation of the Active Metabolite, Endoxifen
Source: J Pers Med. 2023 Jan 31;13(2):272. doi: 10.3390/jpm13020272 (PMC9961245; doi:10.3390/jpm13020272)
Supplement: Supplementary file 1 [file jpm-13-00272-s001.zip › jpm-2113778-supplementary.pdf]

Supplementary Table S1: CYP2D6 Single Nucleotide Variants (SNV) interrogated using the custom open array panel.

**Gene symbol**	**SNP ID (rsID)**	**Variant**	**Allele**
Cyp2D6	rs16947	2850C>T	*2
Cyp2D6	rs769258	31G>A	*35
CYP2D6	rs774671100	137-138insT	*15
CYP2D6	rs35742686	2549delA	*3
Cyp2D6	rs5030867	g.2935A>C	*7
CYP2D6	rs28371706	1023C>T	*17
CYP2D6	rs1065852	100C>T	*10
CYP2D6	rs1135840	4180G>C	*10A,*10B,*10D,*11,*12,*14A,*14B,*17,*19,*20,*21A,*21B,*28,*29,*2A,*2B,*2C,*2D,*2E,*2F,*2G,*2H,*2J,*2K,*2L,*2M,*30,*31,*32,*35,*36,*36,*37,*39,*40,*41,*41A,*41B,*42,*45A,*45B,*46,*47,*49,*4A,*4B,*4C,*4D,*4E,*4F,*4G,*4H,*4K,*4L,*4N,*51,*52,*54,*55,*56A,*56B,*57,*58,*59,*6C,*8
CYP2D6	rs3892097	1846G>A	*4
CYP2D6	rs5030862	124G>A	*12
CYP2D6	rs201377835	883G>C	*11
CYP2D6	rs765776661	4135_4136insTGCCCACTG	*18
CYP2D6	rs267608319	4042G>A	*31
CYP2D6	rs5030656	2615delAAG	*9
CYP2D6	rs72549353	2539_2542delAACT	*19
CYP2D6	rs5030655	1707delT	*6
CYP2D6	rs1080985	-1584C>G	*2A, *21A, *21B, *2A, *2L, *2M, *35, *41, *41A, *42, *45A, *45B, *46, *51, *56A
CYP2D6	rs59421388	3183G>A	*29
CYP2D6	rs28371725	2988G>A	*41
CYP2D6	rs72549346	3259_3260insGT	*42
CYP2D6	rs79292917	2939G>A	*59
CYP2D6	rs5030865	1758G>A	*14A/B
CYP2D6	rs5030865	1758G>T	*8

Subjects' samples were genotyped using a custom panel with variants in Supplementary Table 2. Genotyping was done to select patients with the following *CY2D6* genotypes: CYP2D6*1/*1, CYP2D6*1/*2, CYP2D6*2/*, CYP2D6*1/*17, *2/*17 and *CYP2D6*17/*17*.
